# Near-Infrared Spectroscopy as a Beef Quality Tool to Predict Consumer Acceptance

**DOI:** 10.3390/foods9080984

**Published:** 2020-07-24

**Authors:** Wilson Barragán-Hernández, Liliana Mahecha-Ledesma, Joaquín Angulo-Arizala, Martha Olivera-Angel

**Affiliations:** 1 Centro de Investigación Turipaná, Corporación Colombiana de Investigación Agropecuaria (AGROSAVIA), Montería 230001, Colombia; wbarraganh@agrosavia.co; 2 Facultad de Ciencias Agrarias, GRICA research group, Universidad de Antioquia, Medellin 1226, Colombia; joaquin.angulo@udea.edu.co; 3 Facultad de Ciencias Agrarias, Biogénesis research group, Universidad de Antioquia, Medellin 1226, Colombia; martha.olivera@udea.edu.co

**Keywords:** reflectance, transmittance, meat quality, consumer behavior, discriminant method

## Abstract

This study was conducted to evaluate the feasibility of using near-infrared spectroscopy (NIRS) to predict beef consumers’ perceptions. Photographs of 200 raw steaks were taken, and NIRS data were collected (transmittance and reflectance). The steak photographs were used to conduct a face-to-face survey of 400 beef consumers. Consumers rated beef color, visible fat, and overall appearance, using a 5-point Likert scale (where 1 indicated “Dislike very much” and 5 indicated “Like very much”), which later was simplified in a 3-point Likert scale. Factor analysis and structural equation modeling (SEM) were used to generate a beef consumer index. A partial least square discriminant analysis (PLS-DA) was used to predict beef consumers’ perceptions using NIRS data. SEM was used to validate the index, with root mean square errors of approximation ≤0.1 and comparative fit and Tucker–Lewis index values <0.9. PLS-DA results for the 5-point Likert scale showed low prediction (accuracy < 42%). A simplified 3-point Likert scale improved discrimination (accuracy between 52% and 55%). The PLS-DA model for purchasing decisions showed acceptable prediction results, particularly for transmittance NIRS (accuracy of 76%). Anticipating beef consumers’ willingness to purchase could allow the beef industry to improve products so that they meet consumers’ preferences.

## 1. Introduction

Beef is a high biological value food for human nutrition as a good source of protein, fat, vitamins, and minerals [1]. Regarding the main constituents, beef provides a high quantity, and disponibility of essential amino-acids [2], and an appropriate amount of protein per gram of meal required for a healthy diet [3]. As a source of fat, beef contributes to fatty acids called trans and trans-conjugated fatty acids [4]. Recently, these fatty acids have been related to potential human health improvement, like cancer amelioration [5,6,7]. However, despite these beef properties, consumers are interested in knowing the attributes of the quality of meat, especially intrinsic and extrinsic attributes. Intrinsic attributes refer to the inherent physical characteristics of meat, which cannot be altered without influencing the nature of the product and can be described by the color, visible fat, and appearance of the meat cut. In contrast, extrinsic cues are other factors associated with meat, including the price, brand, label, and production systems [8,9].

Consumer acceptance of beef quality is strongly influenced by visual perceptions and the intrinsic and extrinsic attributes of beef [9,10]. For example, meat color influences freshness perception [10]. A cherry-red color is considered acceptable, but a brown color is not [11], and the consumer will accept or reject a purchase decision based on the color [12]. In addition, as reported by Ardeshiri and Rose [13], consumers can form beef preferences among lighter and darker cherry-red variations.

Frank et al. [14] described the mechanisms associated with the positive effects of intramuscular fat for the beef consumer experience like making food softer, facilitating oral processing, lubricating of food particles, increasing saliva viscosity, and acting as a binder or glue assisting in the formation of a solid bolus in preparation for swallowing. Furthermore, intramuscular fat is positively correlated with tenderness, juiciness, and flavor. However, visible fat has a negative influence on consumers’ quality expectations [9,15]. Morales et al. [16] reported that consumers rated meat as being lower quality when informed of the fat contents but showed a preference for a considerable amount of fat in beef when beef was blindly rated.

Fat is considered to be unhealthy by Mexican consumers, but some fat is necessary to improve beef flavor [17], and Banović et al. [18] demonstrated that Portuguese consumers pay more attention and are more likely to choose meat with red color and lower levels of marbling, similar to the findings reported by Ardeshiri and Rose for Australian beef consumers [6]. On the contrary, Korean beef consumers’ shown preference for beef with high intramuscular fat, and this seems to be related to more juiciness and flavor [7].

Beef’s intrinsic attributes, such as color and fat contents, can be evaluated using a spectrophotometer [19], the Soxhlet method [20], or asking consumers to respond to a questionnaire using a Likert scale [21]. However, these methods could be time-consuming, pollutant, and costly, particularly those related to beef quality.

Near-infrared spectroscopy (NIRS) could be a valuable tool for the rapid quantification and classification of beef qualities for consumer acceptance. Prieto et al. [22] and Weeranantanaphan et al. [23] reported the ability of NIRS techniques to quantify beef quality characteristics. They stated that this technique involves an exact process that can be used for the quantification, classification, and authentication of tasks. However, these reviews did not report the direct application of NIRS to the prediction of beef consumer acceptance.

Over the next 10 years, the Organisation for Cooperation and Development [24] estimates that meat consumption, including beef consumption, will increase, especially in developing countries. As indicated by Realini et al. [25] and Ardeshiri and Rose [13], this situation offers the opportunity for the beef industry to customize products and benefit from beef consumer segmentation. Therefore, the use of NIRS to predict the consumer acceptance of beef intrinsic attributes could represent a valuable tool for beef production chain.

This study was conducted to determine the feasibility of using NIRS techniques to predict visual consumer acceptance and willingness to purchase beef products.

## 2. Materials and Methods

### 2.1. Animals and Sample Collection

The research protocol was approved by the Ethics Committee for Animal Research of Universidad de Antioquia (approval number 116/2018).

Between 15 and 20 steers aged 24 to 36 months were chosen daily, using a convenience sampling method, on nonconsecutive days, until 200 steers had been selected for the final sample. The steers came from fattening production systems characterized by grazing management, a high proportion of naturalized pasture (80% of the area), and low use of concentrate as informed by González-Quintero et al. [26]., The phenotypic characterization was undefined crossbreed between *Bos indicus* and with *Bos Taurus*. Steers were slaughtered in an inspected slaughterhouse in Medellin City, and after two days, meat products were shipped under refrigerated conditions to retail facilities. Carcasses were aged for six days, at 4 °C, and then divided into prime cuts.

### 2.2. Meat Samples

Samples were taken individually (600 g of muscle) from the left longissimus thoracis of each carcass, between the 12th and 13th ribs. Each sample was divided into 5 steaks (25 mm each), trimmed of all subcutaneous fat, photographed before blooming for 20 min, and stored at −80 °C in vacuum-sealed packaging for later analysis.

### 2.3. Beef Steak Images

Photographs were taken using a Canon PowerShot SX60HS camera, equipped with a 33-mm lens that was mounted on a photographic stand to take images of raw steaks. The camera was configured automatically, based on fixed illumination using a D65 fluorescent light [27].

Each photograph was labeled and stored, in both .tiff and RAW formats, for processing with ImageJ^®^ software [28]. Image handling procedures included background removal, size standardization, and residual subcutaneous fat removal from the image.

### 2.4. Development of a Beef Consumer Acceptance Index

Each consumer received a survey, containing a picture and a Likert scale for each intrinsic attribute evaluated. The survey format showed two photographs corresponding to two individual samples, characterized as having either a low or high degree of marbling [29]. Each surveyor rated the photograph for beef color, visible fat (marbling), and overall appearance, using a modified 5-point Likert scale, based on the American Meat Science Association [21] guidelines, to evaluate consumer acceptance, where 1 indicated “Dislike very much”, 2 indicated “Dislike”, 3 indicated “Neither Like or Dislike”, 4 indicated “Like” and 5 indicated “Like very much”. Additionally, to evaluate the consumers’ willingness to purchase the raw beef steaks evaluated in the photographs, the survey asked for the consumers’ disposition to buy the beef, using a dichotomic yes or no answer. The survey also requested information regarding socioeconomic and demographic characteristics for each participant.

The survey sample was established based on population reports [30] and was applied to different socioeconomic levels. A total of 400 surveys were applied, following a proportional stratified sampling method [31,32]. Surveys were applied face-to-face on nonconsecutive days, to allow maximal beef consumer representation [33]. Surveys were performed in retail meat areas, inside of market facilities, to assure that each surveyed participant was a beef consumer [32], and only consumers over 18 years old were chosen. Each survey participant was asked about their willingness to participate in a survey, provided signed informed consent, and received a beef by-product as compensation for participation.

### 2.5. Perception Index: Statistical Analysis and Structural Equation Modeling

The Likert data related to the consumers’ visual perceptions of beef were subjected to polychoric correlation analysis, to ensure that the relationships among Likert variables were >0.3. The Kaiser–Meyer–Olkin measure of sample adequacy (MSA) test was used to verify that variables were pertinent. Likert responses with MSA values < 0.5 were discarded [34]. Factorial analysis was used to develop a consumer acceptance index into a single variable (“visual beef consumer perception index”) [35]. The analysis was developed to apply a maximum likelihood method to factor extraction and used a “varimax” rotation to guarantee factor independence. The construct identified was subjected to the Cronbach’s alpha coefficient, and the factor was validated to ensure it met the criterion of alpha > 0.6 [34]. The visual beef consumer perception index was validated and applied to structural equation modeling (SEM), as indicated by Beaujean [36]. The quality of the model was ensured by the absence of negative variance in the errors, correlations higher than 1, high standard errors, and factorial loadings (FL) > 1. The model was validated to have a root mean square error of approximation (RMSEA) ≤ 0.1, and comparative fit index (CFI) and Tucker–Lewis index (TLI) values ≥ 0.9 [34,36].

All data analysis was performed during index development using a psych [37] and lavaan package [38], in the software R-Project.

### 2.6. NIRS Collection

Each beef sample was subjected to NIRS, collected using reflectance and transmittance. All samples were thawed for 12 h at 4 °C, then subjected to re-blooming for 10 min before pre-processing for spectra collection.

The reflectance method was performed in the Animal Nutrition Laboratory, associated with the Faculty of Agricultural Sciences at the University of Antioquia, using a NIRS DS 2500F monochromator (Foss NIR system, Denmark), with a detector for silicon (800–1100 nm) and lead sulfide (1100–2500 nm). Samples were ground using a refrigerated water mill (IKA M20, IKA-Werke GmbH&Co, Staufen, Germany), to homogenize the sample to a standard particle size. An aliquot of the ground beef sample was collected using a modified syringe, to avoid bubbles [39], and placed in a circular quartz cuvette (70-mm diameter). Subsequently, samples were scanned 40 times, from 800 nm to 2500 nm, at 2 nm increments, and scans were averaged by equipment software (ISI Scan Nova^®^ and MOSAIC^®^) and stored as the reciprocal of reflectance [log (1/R)].

Spectra in transmittance mode were collected at a physicochemical laboratory affiliated with the Zenú food industry research center CI + D, in Medellín (Colombia), using a FoodScan™ (FOSS North America, Eden Prairie, MN, USA). Samples were handled as described by Anderson et al. [40]. Briefly, 180 g of ground, homogenized beef (food processor HC150B 1720 +/−15% rpm), at 10 °C–20 °C, was placed into FoodScan™ sample cups (140-mm diameter × 17.5-mm height). Subsequently, samples were placed in the FoodScan™ chamber, and spectra were obtained in the near-infrared region, from 850 to 1050 nm. Each sample was scanned 20 times, and the scans were averaged by the equipment software and stored as the reciprocal of transmittance [log (1/T)].

### 2.7. Spectra data Analysis

Spectra data manipulation and analyses were performed in the statistical software R-Project [41].

Transmittance and reflectance NIR data were subjected to standard normal variate (SNV) transformation, followed by detrending correction (SNV&D, Barnes, Dhanoa, & Lister [42]). After SNV spectra correction, the first- or second-order derivatives, based on the Savitzky–Golay procedure [43] and using a window size of 5 data points (11 smoothing points), and a second-order polynomial equation, were applied. The spectra transformation and scatter correction were performed using a prospectr package [44].

The mdatools package [45] was used to perform a principal component analysis, using a singular value decomposition algorithm, with a cross-validated process [leave one out (LOO)], to allow two passes for the elimination of outliers, based on H and T statistics. The H statistic assesses how different a sample spectrum is from a population spectra, and the T statistic evaluates how close the predicted value is to the actual value [46].

Partial least square discriminant analysis (PLS-DA) was applied to develop a NIRS prediction model to discriminate the visual beef consumers’ perception index and consumer’s perceptions of beef color, visible fat, and overall appearance, applying a dummy variable. The PLS-DA model allows the covariance between the responses in the Likert categories (Y) and the spectra (X) data matrix to be maximized [43]. In this analysis, the spectra dataset was split into two sets: calibration (70%) and validation (30%). The test set samples were randomly selected, guaranteeing that the samples were uniformly distributed over the spectra variation, to cover the entire variation range in both datasets. The PLS-DA model was generated by the pls package [47], with a nonlinear iterative partial least squares algorithm and LOO cross-validation. The optimum numbers of components in the PLS-DA analysis were retained, based on the relationship between the explained variance among the dependent and independent variables and the calibration and cross-validation processes.

The prediction models developed during the calibration process were validated with the validation dataset. The accuracy prediction during the validation process was assessed by the percentage of samples correctly classified, based on a confusion matrix for the cross-validation process. A ± 0.5 threshold for the correct classification percentage of the predicted Likert category variables was considered [48].

All statistical processes based on the PLS-DA were performed for the original 5-point Likert scale and a simplified Likert scale using three categories. The simplification process resulted from combining Likert categories 1, “Dislike very much” and 2, “Dislike” into a “Dislike” category and combining categories 4, “Like” and 5, “Like very much” into a “Like” category.

## 3. Results and Discussion

### 3.1. Sociodemographic Characteristics

The gender proportions shown in Table 1 were comparable with the proportions reported by the National Administrative Department for Statistics in Colombia (DANE) for the census performed in 2018 [49], where the male proportion was 48.8% and the female proportion was 51.2%. Other characteristics, such as age, income, marital status, and educational status, are presented as general information, due to a lack of Colombian data sources. The proportion of housewives (19.55%) and the proportion of employed (40.58%) participants in this study were comparable with national statistics, but not the proportions of retired (11.49% vs. 3.6%), students (4.11% vs. 18%), and unemployed (0.7% vs. 5.0%) participants. The socioeconomic and demographic characteristics of the sample population were similar to those reported for the national population.

### 3.2. Perception Index

The polychoric correlation among the Likert scale variables revealed a strong agreement between the consumers’ responses to visible fat and color (r_p_ = 0.819, *p* < 0.001), visible fat and overall appearance (r_p_ = 0.883, *p* < 0.001), and color and overall appearance (r_p_ = 0.924, *p* < 0.001). These correlations allowed a cohesive construct to be generated based on visual consumer perceptions. Factor analysis explained 79% of the variance for a single factor. The SEM analysis confirmed the goodness-of-fit for the indexes, CFI = 0.995, TL = 0.984, and RMSEA = 0.1 (Table 2), which met the criteria stated by Kline [50]. Correlations associated with ratings for visible fat and overall appearance could be associated with the consumers’ preferences for low-marbled beef, as indicated by Ardeshiri and Rose [13], as the beef samples used in this study had a median of 2.5% intramuscular fat [29].

To predict the consumers’ visual perceptions, SEM was performed. The results showed the significant contributions (*p* < 0.05) of color and overall appearance, with FL greater than 0.9, and the secondary importance of visible fat and willingness to buy (FL > 0.8). Grunert et al. [8], who used photographs to assess visual beef perception and SEM of 800 consumers in Spain, Germany, France, and the United Kingdom, found that beef color and visible fat showed high loading factors when building their index, but also included price, a parameter that we did not include. Font-i-Furnols and Guerrero [15] also stated that intrinsic cues could be associated with consumers’ expectations relative to past experiences, associating visual beef characteristics with post-purchasing experiences, such as meal preparation and eating conditions, which may motivate the future purchases of the consumer [8].

According to Ngapo et al. [17], Mexican consumers (488), who were characterized as being >37 years old (77%), female (75%), and married (60%), a proportion similar to consumers’ features in the present study, build their beef perceptions based on more than two intrinsic attributes, where color, visible fat, and freshness were the most important. This study found that visible fat and overall appearance affected willingness to purchase.

In contrast to the results of this study, Banović et al. [18] and Morales et al. [16] found that visible fat had a negative relationship with consumer perception.

### 3.3. Estimating Consumers’ Perceptions with NIRS

**Figure 1:**
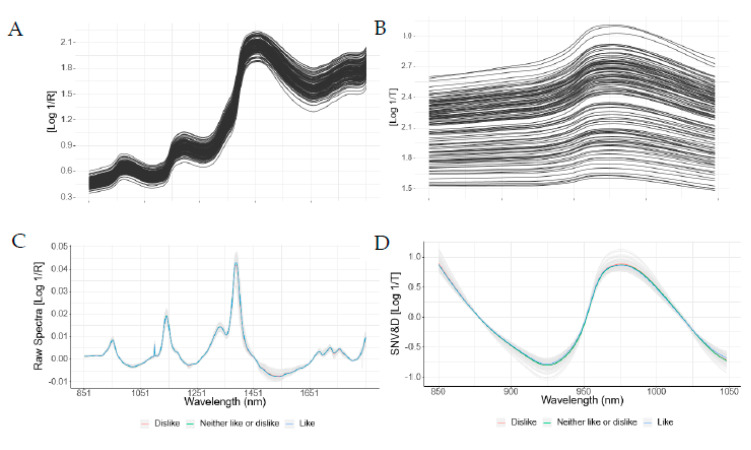
Near-infrared reflectance (**A**) and transmittance (**B**) in the raw spectrum and after mathematical preprocessing for the first derivative (**C**) and SNV&D (**D**) in beef samples.

Raw and preprocessed spectra for the first derivative of reflectance and the SNV&D for transmittance are shown in Figure 1. Both spectroscopy methods presented characteristic absorbance spectra for common organic bonds and near-infrared regions.

The reflectance spectra showed absorbance peaks near 900 nm, related to -OH and -CH bonds, at 1250 nm, associated with the second overtone of -CH, at 1451 nm, related to the first overtone of -OH bonds, and, between 1695 and 1725 nm, linked to the second overtone of -CH bonds. These spectra features are similar to those reported by Cozzolino and Murray [51], Mamani-Linares et al. [52], Prieto et al. [53], and Morsy and Dan-Wen [54]. Similarly, the transmittance spectrum in the present research showed a wide peak between 950 and 1000 nm, related to moisture (934–960 nm and 984–996 nm) and fat (962–968 nm), as indicated by Sierra et al. [55] and De Marchi et al. [56].

The PLS-DA analysis in the visual beef consumer perception assessed by a 5-point Likert scale is shown in Table 3. The calibration process in reflectance spectra to visible fat, overall appearance, and the visual perception index achieved a low percentage of correct classification that ranged from 0% to 68.2%. The Likert scale in consumers’ beef color reached 80% of correct classification in the category “Neither like or dislike”. However, when the consumers’ perception models were externally validated, the maximum rate of correct classification was 77.8%, with accuracy ranging from 29% to 42%. The discrimination of Likert scale in transmittance mode improves the correct classification estimate. The calibration process achieved 70.4%, 82.4%, and 94.1% in the “Neither like or dislike” category in visible fat, overall appearance, and the visual beef perception index, respectively. Nonetheless, the accuracy models ranged between 39.4% and 42.4% for the correct classification during the external validation process.

The simplification of the Likert scale (3-point—Table 4) in visual beef consumers’ perception on the PLS-DA analysis, resulted in a high percentage of correct classification, compared to the 5-point Likert scale. Calibration models for Likert scale prediction in reflectance spectra showed an accuracy of 61.7% in overall appearance and 60.2% in the visible fat. In these models, the Likert categories “Neither like or dislike” and “Like” achieved correct classification percentages of 92.0% and 56.8% in the evaluation of beef visible fat, and 73.3%, and 68% for overall appearance, respectively. Similarly, consumers’ beef color evaluation in the “Neither like or dislike” category reached 80% correct classification. However, the calibration color model obtained a low model accuracy (30.4%).

Furthermore, the visual beef consumers’ perception index followed a similar behavior to that observed in the visual color calibration model. The performance of the transmittance model on a 3-point Likert scale reached the highest accuracy in the calibration and external validation, with 73.2% and 54.6% in the visible fat. Besides, external validation models achieved an accuracy lower than 53%.

The prediction of consumers’ perceptions of visible fat by NIRS in transmittance mode achieved the highest correct classification rate among the intrinsic attributes evaluated, with accuracy values of 73.2% and 54.6% during the calibration and external validation models, respectively, when using the simplified 3-point Likert scale. This model showed a high rate of error (ranging from 33.4% to 83.3%). However, for the prediction of willingness to purchase, the accuracy during the external validation process increased to 76% (Table 4). No other studies have been performed examining this outcome for comparisons.

The abilities of reflectance and transmittance NIRS to predict the intrinsic attributes of meat [22], and meat classifications during the adulteration/authentication process [57,58], have been widely documented. In comparison, the ability of NIRS to predict consumer sensory parameters related to beef has been less well documented, and contradictory results have been reported [59]. Until now, no other studies have examined the application of NIRS for the prediction of consumers’ visual perceptions of beef attributes.

The research reported by Liu et al. [60] regarding NIRS for the prediction of beef sensory attributes used a trained sensory panel (*n* = 7) to evaluate how chewiness and juiciness correlated with reflectance data and reported R-squared (*R*^2^) calibration values of 0.58 and 0.5, respectively, concluding that NIRS data had minimal predictive abilities. Furthermore, Yancy et al. [61] applied reflectance NIRS to predict beef consumers’ (*n* = 240) perceptions in a visual- and taste-based experiment, examining the overall impression and tenderness of beef, and reported a predictive model with *R*^2^ values during the full cross-validation (*R*^2^_cv_) process of 0.79 and 0.74, respectively.

Recently, Magalhães et al. [62] applied NIRS reflectance data, obtained from intact *longissimus thoracic* muscle (*n* = 644) from Nelore steers, to predict meat quality attributes closely associated with beef consumers’ acceptance, such as share force, marbling, and *CIELab* color. They reported a low predictive ability (*R*^2^ prediction values from 0 to 0.45), associated with high beef heterogeneity, especially for the marbling score. Likewise, Cafferky et al. [63] reported the low accuracy of NIRS reflectance predictive models for beef Warner–Bratzler shear force (*R*^2^_cv_ = 0.14–0.22), sensory texture (*R*^2^_cv_ = 0.06–0.13), sensory juiciness (*R*^2^_cv_ = 0.04–0.09), and sensory flavor (R^2^_cv_ = 0.04–0.28), as assessed by a trained sensory panel (*n* = 12). On the contrary to the above-cited negative reports, Prieto et al. [64], showed successful application of NIRS (1100–2500 nm) to the prediction of beef’s physical parameters using ground meat, arguing that correlation between color measurement (L*, a* and b*) and intramuscular fat, allowed to the developed of the NIRS prediction model.

The limited ability of NIRS to predict meat quality attributes preferred by beef consumers has been associated with the narrow scale used to evaluate sensory characteristics, and high heterogeneity among the meat traits within muscle contributes to the generation of significant bias within NIRS models [59]. In addition, Liu et al. [60] stated that handling processes, such as freeze-thaw, could contribute to samples scanned by NIRS having different attributes from those tested by consumers and trained panels or analyzed in laboratories. Moreover, Cozzolino and Murray [65] and Prieto et al. [48] affirmed that the absence of concordance between NIRS spectra and physical beef attributes could be related to the grinding process. Grinding affects muscle integrity by altering the form, which alters how light is trapped and scattered in the muscle and disrupts the randomized muscle fiber arrangement, which alters light scattering. On the other hand, Ripoll et al. [66] reported accurate NIRS prediction models for instrumental beef characteristics like Warner–Bratzler (*R*^2^_p_ = 0.743) and water holding capacity (*R*^2^_p_ = 0.829), and the sensory attribute tenderness (*R*^2^_p_ = 0.981), using ground meat. These authors state that indirect spectral information, retrieved from beef moisture and fat and their relationship with physical beef parameters, allowed the development of the prediction models for instrumental and sensory beef parameters.

The PLS-DA used to predict consumers’ willingness to purchase beef, as evaluated by assessments of beef photographs (Table 4), showed accuracy values of 69.6% and 75.6% during calibration and 70% and 75.8% during external validation for the reflectance and transmittance models, respectively. Both models showed a high percentage of correct classification for positive disposition to buy, ranging from 93% to 100%. However, the correct classification of negative decisions only reached 33% for the transmittance model.

**Figure 2:**
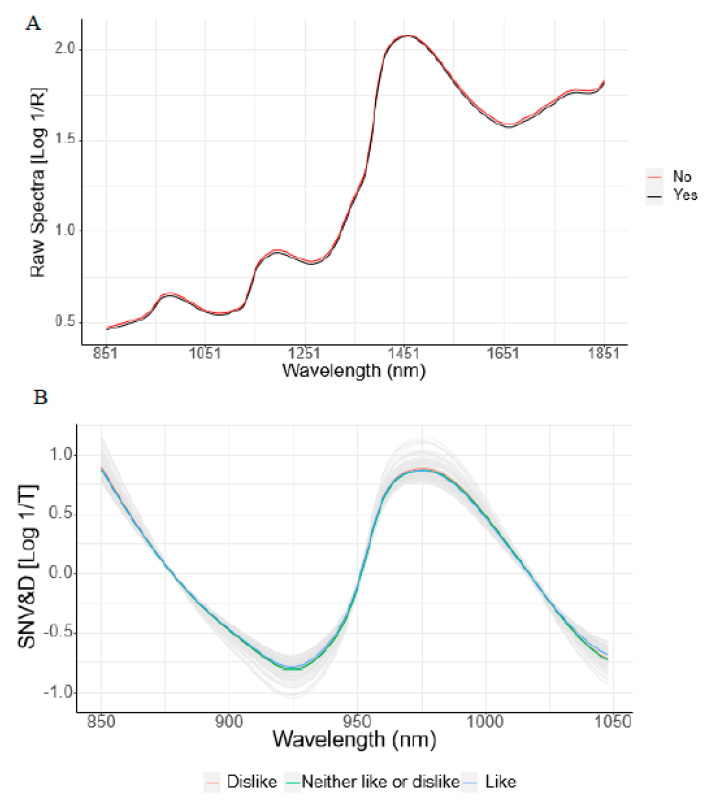
Near-Infrared reflectance (**A)** raw spectra and transmittance (**B**) standard normal variate (SNV) spectra for consumer’s purchasing decisions of beef samples.

The average NIRS spectra of both reflectance and transmittance methods for willingness to buy are shown in Figure 2. Both spectra modes showed that the spectra of samples that were negatively rated to buy reached the highest absorbance at 950 nm, 1250 nm, and between 1695 and 1725 nm for reflectance, and from 962 to 968 nm in transmittance. These wavelengths were related to -CH molecular bonds [43,44,45,46,47,48]. Similar to the statement by Ripoll et al. [66], the spectra fingerprint related to visible fat in the beef sample used for this study could have contributed to separating the unfavorable decision to buy associated with more visible fat, from lean beef samples.

Ardeshiri and Rose [13] affirmed that fat and marbling played important roles in purchasing decisions. Consumers use these beef attributes to create a perception of beef quality, integrating past experiences and future expectations [9,15]. Therefore, the relationship between beef visual attributes and consumer’s purchasing decisions in the present study could explain the better performance of the beef purchasing discrimination model, especially for transmittance data, where spectra separation in the near-infrared transmittance region associated with fat could be observed [55].

## 4. Conclusions

We proposed a beef consumers’ perception index, based on the evaluation of beef’s intrinsic attributes, supported by a high correlation among beef color, visible fat, and overall appearance, and generated using an SEM model.

The transmittance spectral data could be used to develop an acceptable discrimination model for consumers’ purchasing decisions using ground meat. Our results reported an accuracy of 76%, but these models could be improved by the use of NIRS equipment robust enough to read intact samples.

The prediction of consumer’s willingness to purchase could improve the marketing dynamic in the beef production chain, allowing beef producers and animal science technicians to develop a production strategy to produce beef with a high probability of purchase.

In the meat industry, the NIRS prediction model of consumers’ willingness to buy beef would allow the segmentation of meat with potential to be purchased, from meat that would not be purchased and would be assigned for the production of meat by-products.

## Figures and Tables

**Table 1:** Socioeconomic and Demographic Characteristics of the Survey Participants.

Sample (*n* = 400)		
Gender	Male	41.32%
	Female	58.67%
Age (years)	<37	31.78%
	37–55	38.14%
	55–74	28.11%
	>74	1.95%
Income	Undeclared	16.38%
(times the statutory minimum wage)	<1	11.49%
	1–2	41.07%
	3–4	21.02%
	5–6	7.33%
	>6	2.68%
Marital status	Married	43.27%
	Separated	6.35%
	Single	30.56%
	Widow	3.66%
	Consensual union	15.64%
	Other	0.4%
Educational status	Primary school	8.55%
	Secondary school	35.94%
	Technological level	21.51%
	Professional	22.00%
	Postgraduate	11.00%
	Other	0.90%
Employment	Housewife	19.55%
	Unemployed	0.7%
	Employed	40.58%
	Retired	11.49%
	Student	4.15%
	Self-employed	23.47%

**Table 2:** Factor analysis (varimax rotation) and structural equation model for visual beef consumer perception.

Likert Variable ^1^	Communality	Cronbach’s Alpha	KMO ^2^	Beef Consumers Perception ^3^	Variance
Color	0.90	0.894	0.85	0.88	79%
Visible fat	0.84		0.85	0.83	
Overall appearance	0.98		0.76	0.99	
Willingness to buy	0.84		0.89	0.84	
Perception Index	Independent Variable		Factor Loading (FL)		*p*-value
Visual beef consumers’ perception	Color		0.902		<0.001
	Visual fat		0.841		<0.001
	Overall appearance		0.975		<0.001
	Willingness to buy		0.843		<0.001

^1^ Likert scale response (1. “Dislike very much” and 5. “Like very much”). ^2^ The Kaiser–Meyer–Olkin measure of sampling adequacy. ^3^ Factor loadings.

**Table 3:** Partial least square discriminant analysis for visual beef consumer perception (5-point Likert scale) based on transmittance and reflectance near-infrared spectra.

		Reflectance				Transmittance			
		Mat. Process. ^1^	C ^2^	Calibration ^3^ (%)	External Validation ^3^ (%)	Mat. Process.	C	Calibration ^3^ (%)	External Validation ^3^ (%)
Color	1. Dislike very much	Raw	3	0.000	0.000	SNV&D	7	8.30	0.000
	2. Dislike			14.30	16.70			45.50	0.000
	3. Neither like or dislike			80.00	75.00			64.70	33.30
	4. Like			29.70	18.80			46.90	76.90
	5. Like very much			0.000	0.000			0.000	0.000
	Model accuracy			28.40	32.50			38.10	40.00
Visible fat	1. Dislike very much	First derivative	3	0.000	0.000	Second derivative	2	0.000	0.000
	2. Dislike			25.00	0.000			14.30	0.000
	3. Neither like or dislike			60.00	28.60			60.90	77.80
	4. Like			67.90	70.00			70.40	54.50
	5. Like very much			25.00	0.000			0.000	0.000
	Model accuracy			47.60	29.00			41.50	39.40
Overall appearance	1. Dislike very much	SNV&D	5	10.00	0.000	SNV&D	7	0.000	0.000
	2. Dislike			14.30	0.000			25.00	33.30
	3. Neither like or dislike			60.00	77.80			82.40	57.10
	4. Like			62.50	60.00			54.30	53.30
	5. Like very much			0.000	0.000			0.000	20.00
	Model accuracy			43.90	39.40			42.70	42.40
Visual Perception index	1. Dislike very much	Raw	7	0.000	0.000	SNV&D	4	0.000	0.000
	2. Dislike			22.20	0.000			0.000	0.000
	3. Neither like or dislike			68.20	66.70			94.10	57.10
	4. Like			56.30	55.00			56.40	56.30
	5. Like very much			0.000	0.000			0.000	0.000
	Model accuracy			43.10	42.50			45.80	40.60

^1^ Mathematically preprocessed. ^2^ Number of components in the PLS-DA model. ^3^ Percentage of correct classification.

**Table 4:** Partial least square discriminant analysis for visual beef consumer perception index (3-point Likert scale) and consumers’ willingness to purchase based on transmittance and reflectance near-infrared spectra.

		Reflectance				Transmittance			
		Mat. Process. ^1^	C ^2^	Calibration ^3^ (%)	External Validation^3^ (%)	Mat. Process.	C	Calibration ^3^ (%)	External Validation ^3^ (%)
Color	1. Dislike	SNV	3	0.00	0.00	SNV	7	17.40	12.50
	2. Neither like or dislike			80.00	83.30			76.50	66.70
	3. Like			28.80	27.80			50.00	64.70
	Model accuracy			30.40	37.50			46.40	51.60
Visible fat	1. Dislike	First derivative	7	13.30	20.00	SNV&D	10	35.70	16.70
	2. Neither like or dislike			92.00	57.10			82.60	55.60
	3. Like			56.80	57.90			80.00	66.70
	Model accuracy			60.20	51.60			73.20	54.60
Overall appearance	1. Dislike	Second derivative	5	29.40	14.30	First derivative	7	16.70	16.70
	2. Neither like or dislike			73.30	66.70			76.50	71.40
	3. Like			68.00	41.20			63.80	55.00
	Model accuracy			61.70	43.80			56.10	51.50
Visual perception index	1. Dislike	First derivative	6	0.00	14.30	SNV	7	11.10	16.70
	2. Neither like or dislike			72.70	77.80			76.50	71.40
	3. Like			57.60	50.00			52.10	57.90
	Model accuracy			49.0	50.00			48.20	53.10
Willingness to buy	No	Raw	1	0.00	0.00	SNV	4	33.30	33.30
	Yes			100	100			93.10	91.70
	Model accuracy			69.60	70.00			75.60	75.80

^1^ Mathematically preprocessed. ^2^ Number of components in the PLS-DA model. ^3^ Percentage of correct classification.
